# Self-management support for chronic disease in primary care: frequency of patient self-management problems and patient reported priorities, and alignment with ultimate behavior goal selection

**DOI:** 10.1186/s12875-019-1012-x

**Published:** 2019-08-29

**Authors:** Danielle M. Hessler, Lawrence Fisher, Vicky Bowyer, L. Miriam Dickinson, Bonnie T. Jortberg, Bethany Kwan, Douglas H. Fernald, Matt Simpson, W. Perry Dickinson

**Affiliations:** 1 Department of Family & Community Medicine, University of California-San Francisco, San Francisco, CA 94143 USA; 2 Department of Family Medicine, University of Colorado School of Medicine, Aurora, CO USA

**Keywords:** Self-management, Shared decision making, Goal setting, Chronic disease

## Abstract

**Background:**

To enable delivery of high quality patient-centered care, as well as to allow primary care health systems to allocate appropriate resources that align with patients’ identified self-management problems (SM-Problems) and priorities (SM-Priorities), a practical, systematic method for assessing self-management needs and priorities is needed. In the current report, we present patient reported data generated from Connection to Health (CTH), to identify the frequency of patients’ reported SM-Problems and SM-Priorities; and examine the degree of alignment between patient SM-Priorities and the ultimate Patient-Healthcare team member selected Behavioral Goal.

**Methods:**

CTH, an electronic self-management support system, was embedded into the flow of existing primary care visits in 25 primary care clinics and was used to assess patient-reported SM-Problems across 12 areas, patient identified SM-Priorities, and guide the selection of a Patient-Healthcare team member selected Behavioral Goal. SM-Problems included: BMI, diet (fruits and vegetables, salt, fat, sugar sweetened beverages), physical activity, missed medications, tobacco and alcohol use, health-related distress, general life stress, and depression symptoms. Descriptive analyses documented SM-Problems and SM-Priorities, and alignment between SM-Priorities and Goal Selection, followed by mixed models adjusting for clinic.

**Results:**

446 participants with ≥ one chronic diseases (mean age 55.4 ± 12.6; 58.5% female) participated. On average, participants reported experiencing challenges in 7 out of the 12 SM-Problems areas; with the most frequent problems including: BMI, aspects of diet, and physical activity. Patient SM-Priorities were variable across the self-management areas. Patient- Healthcare team member Goal selection aligned well with patient SM-Priorities when patients prioritized weight loss or physical activity, but not in other self-management areas.

**Conclusion:**

Participants reported experiencing multiple SM-Problems. While patients show great variability in their SM-Priorities, the resulting action plan goals that patients create with their healthcare team member show a lack of diversity, with a disproportionate focus on weight loss and physical activity with missed opportunities for using goal setting to create targeted patient-centered plans focused in other SM-Priority areas. Aggregated results can assist with the identification of high frequency patient SM-Problems and SM-Priority areas, and in turn inform resource allocation to meet patient needs.

**Trial registration:**

ClinicalTrials.gov ID: NCT01945918.

## Background

Managing a chronic health condition, like type 2 diabetes, requires many ongoing patient activities occurring in parallel, e.g., eating a healthy diet, regular physical activity, and taking medications as directed [1, 2]. The sheer number of tasks can be overwhelming for individuals, with many of the required changes for many viewed as difficult to achieve [1, 3]. Given that almost all daily management decisions are made by patients outside of a healthcare setting with < 1% of an individuals’ time spent with their healthcare team [4], it is crucial to foster patient engagement and persistence in managing their chronic illness to achieve desired clinical goals [5, 6].

Self-Management Support (SMS) refers to the process of education and support provided to people with chronic health conditions and their families to help them understand their central role in managing their disease, to make informed decisions about care, and to engage in healthy behaviors [7–9]. While recognizing that patients often have several competing self-management needs (e.g., diet, exercise, stress, substance use, medication taking), in practice specific areas of patient self-management support are often siloed (e.g., nutrition or exercise), rather than prioritized in a meaningful way. Furthermore, clinicians and other members of the healthcare team often are unaware of patient priorities and resources, making it hard to align patient needs with clinician/healthcare team preferences [10, 11]. Related literature on shared decision-making points to the importance of considering patient priorities and collaboratively setting goals to enhance and sustain behavior change [12, 13]. However, only a small minority of patients are routinely asked about their self-management behaviors or preferences [9, 14, 15]. Furthermore, when screening of self-management behaviors does occur, it typically does not address the full range of required disease self-management behaviors and occurs with limited feedback to patients; which in turn constrains comprehensive collaborative care planning. For example, in Krist and colleagues’ review, they note that only 10–20% of smokers report being told to quit smoking by their clinician and less than 20% of obese patients report being told by their clinician that they are overweight [14, 15].

To enable delivery of high quality patient-centered care, as well as to allow primary care health systems to allocate appropriate resources that align with patients’ identified self-management problem areas and priorities, a practical, systematic method for assessing self-management needs and priorities is needed. Connection to Health (CTH) is an electronic SMS system for primary care that addresses these problems by recording patients’ self-management problems and challenges across a range of areas, assessing patient priorities, and facilitating structured action planning and follow-up through a process of shared goal setting (see www.conntectiontohealth.org to learn more). Thus, CTH provides a useful method for gathering information from patient cohorts about disease self-management in primary care. Such information can be useful for identifying high frequency self-management problems and patient priority areas, and in turn inform needs for adequate resources and care plans to address these needs and priorities.

In the current report, we utilize CTH data generated from patients with a chronic disease in 25 primary care clinics to further explore the following questions to enhance our understanding of SMS provision and gaps in primary care settings for patients with chronic disease: (1) Which areas of self-management do patients with chronic disease report the most difficulty?, (2) Which areas of self-management do patients tend to prioritize for behavior change? (3) Which areas of self-management do patients and Healthcare team members ultimately select as goals or targets of behavior change? (4) To what extent do these goals align with initial patient priorities?

## Methods

### Design

This report focuses exclusively on the patient-reported health assessment and priority-based goal setting data collected as part of a three-arm, *cluster-randomized trial* to evaluate strategies for implementing CTH in diverse primary care practices.

### Practice sample

Twenty five primary care practices, 13 in Colorado and 12 in Northern California participating in the CTH study were randomized to the two study arms utilizing the electronic CTH program. Inclusion criteria for practices were family medicine or general internal medicine practices with a minimum of 80 patients with T2DM. A diverse set of practices of various sizes and organizational structures were recruited (i.e., private, system-owned, and safety net practices). Practices participated for 18 months, with participation encompassing December 2013 to March 2017 [16].

### Procedure

**Fig. 1:**
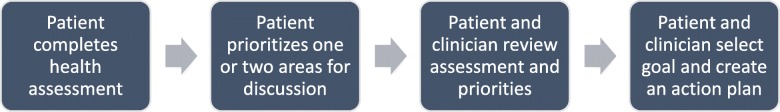
Connection to Health Patient Flow

The CTH program guides patients and members of the healthcare team through the following steps (Fig. 1): (1) patient assessment to identify self-reported management problems (SM-Problem), (2) identification of patient-reported management priorities (SM-Priorities), and (3) creation of an action plan and follow up process with a member of the healthcare team to select a self-management goal. In face to face meetings with a member of their healthcare team, patients were introduced to the program through an iPad logged into the CTH system. Patients were asked to complete a web-based, electronic assessment of their current self-management in twelve SMS areas. Based on national guidelines and validated scoring, automated algorithms flagged problem areas for patients and a member of the healthcare team, with recommendations for change. After patients reviewed a summary of their assessment results, the program prompted them to prioritize up to two areas they wished to discuss with a member of the healthcare team. Patients and a member of the healthcare team then met to review the assessment results, select a goal and develop a subsequent action plan to achieve the goal.

Given the overall aims of the larger study, clinics generally focused on working with patients with type 2 diabetes, although they were also encouraged to utilize CTH with patients diagnosed with other chronic illnesses. There were no patient eligibility criteria. As part of the study design, all practice members (clinicians and staff) participated in two hours of training including an introduction to SMS and an interactive tutorial on the CTH system including a practice dyad using the summary report and engaging in goal setting. Practices selected the subset of healthcare team members to use CTH in patient encounters. The majority (> 90%) of those team members identified as community health workers (CHWs), health educators, panel managers, chronic care managers, volunteers, or similar roles; while a small portion identified as a nurse or Certified Diabetes Educator (CDE) or primary care physician. The research protocol was approved by the University of Colorado at Denver and the University of California, San Francisco institutional review boards who waived any patient consent given the data presented were collected and used at the point of care and provided to the research team in de-identified form.

### Measures

Congruent with policy recommendations from the Society of Behavioral Medicine [17], the CTH assessment utilized brief scales that are reliable, sensitive to change, and age appropriate to assess 12 areas of patient self-management [18]. Each area of self-management was automatically scored based on national guidelines with results for each presented in three categories: Green (no need for change), Yellow (moderate level/need for change), and Red (high level/need for change). Areas of SMS, items and cut-points for scoring appear in Table 1 for each of the twelve SMS areas including: BMI (based upon self-reported height and weight), diet (servings of fruits and vegetables, salt, fat, sugar sweetened beverages [19–21], physical activity (frequency and duration of weekly moderate to strenuous physical activity; [22], missed medications (number of days missed in past 7 days; [23–25] tobacco and alcohol use (frequency of use and binge drinking; [26–29], health-related distress (5 items based on the Diabetes Distress Scale, [30, 31] presence of a recent major life stressor) [32], and depression symptoms (assessed with the PHQ8); [33, 34]. Frequency of each of the 12 areas by category (green, yellow, red) along with priorities for discussion and ultimate self-management goal were recorded. Additional patient demographics (age group, gender, race, education level, and reported chronic diseases) were additional captured to describe the sample.

**Table 1:** Description of Self-management assessment areas and algorithm for scoring responses

Health Area	Questions	Level of Concern		
		Green (low risk)	Yellow (moderate risk)	Red (greater than moderate risk)
BMI	Height and weight	BMI < 25	BMI 25–29.9	BMI ≥30.0
Health Distress	Stress related to living with and managing health problems over past month. Five items on scale from “not a problem” (1) to “a very serious problem” (5).	Score < 3 on all items	*No category*	Score of ≥3 on any item
Fat Intake	Number of days, and typical portion size, of regular fat foods from a fast food restaurant consumed over the past week	0 days or 1 day /any portion size	2 days/small or medium portions; or 3 days/small portion	2 days/large or supersize; or 3 days/medium, large or supersize portions; or ≥ 4 days/any portion
Fruit and Vegetable Intake	Number of servings of fruits and vegetables consumed in a usual day over the past week	≥5 servings	3 to 4 servings	≤2 servings
Sugared Beverage Intake	Number of 12-oz sodas /other sugar-sweetened drinks per day over past week	No drinks	1 to 2 drinks	≥3 drinks
Salt Intake	Number days canned, processed, or pickled foods consumed over the past week, and addition of extra salt to food or in cooking	≤ 2 days and no extra salt added to food or in cooking	*No category*	3 days, and/or extra salt was added to food or in cooking
Physical Activity	Number of minutes of physical activity over the past week	≥150 min	*No category*	≤150 min
Medication Adherence	Number of days in past week that one or more medication doses was missed	0 days	*No category*	≥1 day
Alcohol Intake	Number of alcoholic drinks consumed in the past week, and binge drinking (consuming more than 3 drinks (female) or 4 drinks (male) on any day in the past month)	≤ 14 weekly drinks (men < 65), 7 weekly drinks (women and men ≥65) & no binge drinking	*No category*	≥15 weekly drinks (men < 65), 8 weekly drinks (women and men ≥65), OR binge drinking
Tobacco Use	Current tobacco use in the past week	Not a tobacco user	*No category*	Current tobacco user
Depression Symptoms	Depression symptoms over the past two weeks (Patient Health Questionnaire depression scale; PHQ-8)	Score ≤ 9	Score 10 to 14	Score ≥ 15
General Life Stress	Stress around major life events in the past week (e.g., family, work)	Endorses no to general life stress	*No category*	Endorses yes to general life stress

### Data analysis

Descriptive statistics were computed to review item and scale distributions and frequencies by health area. Chi-square tests or *t* tests, as appropriate, tested for differences between completers of priority and goal setting versus non-completers and associations with patient characteristics. Frequencies of SM-Priorities were examined contingent upon the presence of a SM-Problem in that area. Goals selected during action planning were examined in cross-tabs to document frequency of agreement. Generalized Linear Mixed Models (GLMM) examined frequencies adjusting for clustering by clinic as well as patient characteristics associated with the outcomes of interest. Sensitivity analyses using mixed models examined select planned patient subgroups for select common chronic diseases with the same adjustment factors. Data were analyzed using SPSS v.19 software.

## Results

The majority of the 25 participating clinics were Community Health Centers (*n* = 18; 72%), with remaining split between independent practices or practices that were part of an integrated healthcare system; and 16 (64%) had achieved NCQA PCMH recognition. Clinics employed on average 6.8 (+ 3.7) FTE clinicians and served 37% (+ 20%) patients on Medicaid.

A total of 446 patients across the 25 clinics completed the health assessment (*n* = 287 for Colorado and *n* = 159 for California clinics). Participants were on average 55.4 (+ 12.6) years of age, 58.5% were female, and 52.6% had an education level < a high school diploma (Table 2). The majority identified themselves as Hispanic (42.6%) or non-Hispanic white (40.1%). In line with expectations for primary care, the most common chronic illnesses reported included diabetes (67.5%), hypertension (58.5%), and hypercholesterolemia (55.8%). All patients identified themselves as having at least one chronic illness, with 78.5% identifying two or more co-morbid chronic illnesses. Three-quarters of the sample (*n* = 336) prioritized one (*n* = 85) or two (*n* = 251) areas of self-management to discuss with their healthcare team (SM-Priorities). Of these, 74.7% (*n* = 227) went on to select a self-management goal and to create an action plan for change. Key reasons reported by member of the healthcare team for patients not completing action plans were clinic-based – primarily insufficient time due to work flow. There were no significant differences between patients who did not prioritize health areas or did not create an action plan compared to those who did on the basis of any assessed patient characteristic, reported health condition, or type of self-management need.

**Table 2:** Patient characteristics

	*n* = 446 Mean (SD) or % (n)
Age (years)	55.4 (12.6)
Gender (% female)	58.5% (261)
Race (%)	
American Indiana or Alaskan Native	0.2% (1)
Asian	6.7% (30)
Black or African American	3.8% (17)
Native Hawaiian / Pacific Islander	1.3% (6)
White	40.1% (179)
Hispanic	42.6% (190)
Multi-ethnic background	3.6% (16)
Education	
Less than High School	21.1% (94)
High School or GED	31.6% (141)
Some College	25.8% (115)
College or Higher	18.6% (83)
BMI	33.9 (8.1)
Number of Chronic Conditions	
One	21.5% (96)
Two	31.6% (141)
Three or more	46.9% (209)
Chronic Conditions	
Diabetes	67.5% (301)
Pre-diabetes	18.2% (81)
Hypertension	58.5% (261)
Hypercholesterolemia	55.8% (249)
Asthma	13.9% (62)
COPD or emphysema	6.5% (29)
Cardiovascular disease	10.3% (46)
Other chronic condition	16.8% (75)

Note: A small portion of the sample did not report on race (1.6%) and education level (2.9%)

### Self-management problem areas (SM-problems)

**Fig. 2:**
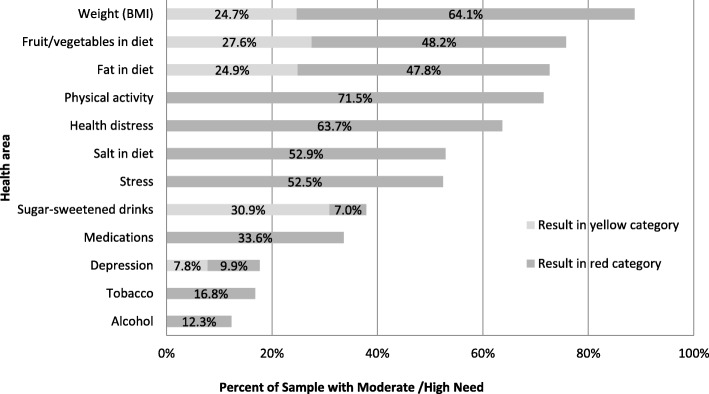
Frequency of participant needs by health area (n = 446)

The frequency of patient-identified SM-Problems for each of the 12 areas is summarized in Fig. 2, with the most frequent areas targeting weight loss (88.8%), eating more fruits or vegetables (75.8%), reducing dietary fat (72.6%), increasing physical activity (71.5%), and high levels of health-related distress (63.7%).

Of the 12 SM-Problem areas, on average patients reported 6.0 (+ 2.1) areas of need for change (red) and 1.2 (+ 1.0) areas of moderate need for change (yellow). No patients reported zero SM-Problem areas, and only 1.3% reported one SM-Problem area. Thus, the average number of reported problem areas per patient was quite high.

### Patient-reported priorities for self-management (SM-priorities)

**Fig. 3:**
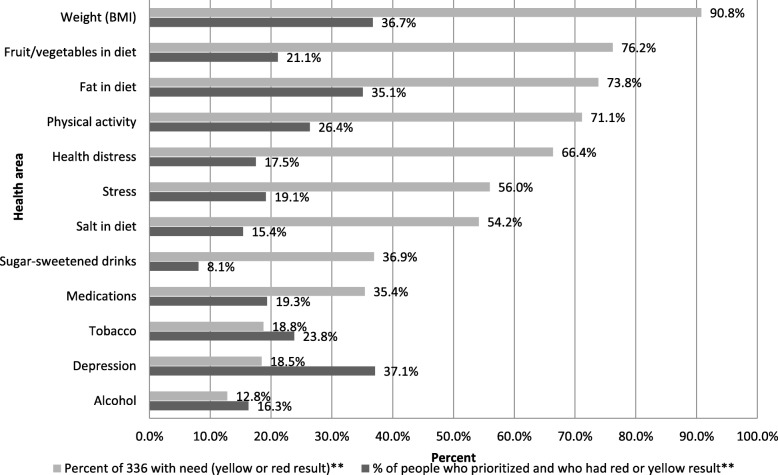
Frequency of patient priority areas contingent on need (n = 336 for individuals with priorities)

Frequencies of patient-selected SM-Priorities, among those endorsing a given a SM-Problem, are displayed in Fig. 3. The frequency of prioritized SMS problems was highly variable across the 12 areas: the most frequent were weight loss (36.7%), fat in diet (35.1%), and depression symptoms 37.2%; the least frequent were decreasing sugar-sweetened drinks (8.1%), salt (15.4%), alcohol (16.3%) and health-related distress (17.5%). Considering rates of needs and priorities in tandem, it is interesting to note that weight loss and lowering fat in diet were self-management areas that were frequently endorsed by patients as both a SM-Problem and SM-Priority. Notably, although health-related distress was reported as an SM-Problem by over two-thirds of the sample, it was only identified as an SM-Priority for 17.5% of those individuals. Likewise, while three-quarters of participants reported a need for additional fruits/vegetables in their diet, only 21.1% of these individuals prioritized this area.

### Goal setting and alignment with patient-reported SM-priorities

**Fig. 4:**
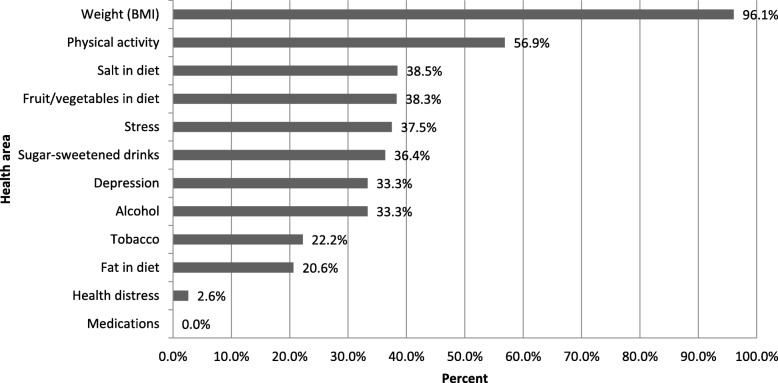
Percent of prioritized health areas selected as action plan goals with HCP by health area

Overall, 72.7% of the sample created an action plan with a member of the healthcare team that focused on a goal in line with one of the patient-selected SM-Priorities. However, the frequency of alignment between action plan goals and patient SM-Priority areas varied greatly depending on SM-Problem area (Fig. 4). Of the patients who created action plans with an SM-Priority of weight loss (*n* = 76), almost all (96%) went on to create an action plan goal specifically targeting their weight or a behavior intended to directly result in weight loss (physical activity, specific changes to diet). Patient SM-Priorities of physical activity (*n* = 51) were also frequently translated into action plans that focused on physical activity (56.7%). However, concordance between patient SM-Priorities and their ultimate action plan goals was considerably lower for the remaining SM-Problem areas (Fig. 4). Strikingly, of the patients who prioritized health-related distress and medication taking, virtually none developed an action plan with their HCP that targeted these areas (2.6 and 0% respectively). When action plan goals did not align with any patient SM-Priorities, goals were typically made around physical activity, weight loss or diet. Of the patients who prioritized health-related distress, 50% made a diet/weight loss goal and 26% made a physical activity goal; with similar results for those who prioritized medication taking (56% made a diet/weight goal and 19% made a physical activity goal).

### Additional analyses

There was no consistent pattern of association between any of the recorded patient demographic factors (age, gender, race, education level) and the measures of interest including the frequency of SM-Problems and SM-Priorities or alignment between SM-Priorities and Goal selection with the exception of patient age. Compared to older adults (age 40–64, 65 or older), younger adults (19–39 years) were more likely to report SM-Problems including: missed medications (24.0 and 33.6% vs. 51.0% *p* = .004), high levels of alcohol use (10.4 and 7.3% vs. 33.3% *p* < .001), and tobacco use (18.4 and 6.3% vs. 27.5%, *p* = .002). In addition, stress was elevated for the 19–39 year old (58.8%) and 40–64 year old groups (56.2%) relative to the oldest age group (37.5%, *p* = .004). Adjusting for clustering by clinic and patient age, virtually identical results were found for the frequency of SM-Problems, SM-Priorities, and degree of alignment with Goals. Likewise, analyses limited to each of the three most common chronic illnesses (diabetes, hypertension, and hypercholesterolemia) yielded similar results.

## Discussion

This study assessed patient reported SM-Problems, SM-Priorities, and Patient-Healthcare team member Goal Selection. The current findings suggest that patients with chronic disease in primary care experience multiple competing SM-Problems. On average, patients reported experiencing simultaneous challenges in 7 out of the 12 SM-Problems, with less than 2% of patients reporting only one single SM-Problem. In line with related literature, the frequency for several SM-Problems was elevated among relatively younger adults living with chronic disease (age 19–39 years) [35, 36]. Patient SM-Priorities were variable across the self-management areas with the most frequently prioritized areas being weight loss, decreased fat in diet, and depression symptoms. Ultimate Patient-Healthcare team member Goal selection were aligned well with patient SM-Priorities when patients prioritized weight loss or physical activity, but suggested potential misalignment in other self-management areas, such as stress/health-related distress, medication taking, and tobacco/substance use.

The current findings suggest that patients with chronic disease in primary care are able to report on their self-management behaviors and to prioritize them among their often competing self-management problems or areas of challenge. In aggregate, this type of data can assist with the identification of high frequency patient SM-Problems and SM-Priority areas, and in turn to inform resource allocation as well as opportunities to better understand why patients generally are not viewing specific SM-Problems as a priority. Second, findings comparing ultimate goal selection to SM-Priorities illustrate areas of alignment vs. potential misalignment and missed opportunities for using goal setting to create targeted patient-centered plans. The lopsided use of goal setting for the inter-related areas of weight loss, diet, and physical activity is not surprising given the roots of action planning that have traditionally focused on these behavioral lifestyle elements [37]. However, results suggest that even when presented with alternative patient SM-Priorities, members of the healthcare team by and large do not expand their use of goal setting or planning to these other areas of self-management, such as stress/health-related distress, medication taking, and substance use. Findings can inform opportunities and discussion for creating greater alignment with patient priorities in these self-management areas.

Viewing patients’ SM-Problems, SM-Priorities and ultimate goal selection together, several notable patterns emerge. Weight loss and physical activity emerged as the most common areas of patient SM-Problems, with high rates of patient SM-Prioritization and translation into action plan goals. This concordance, however, did not extend beyond these two areas: in contrast, health-related distress, conceptually distinct from depression and more closely tied with chronic disease health outcomes [38, 39], was one of the most common SM-Problems, but was chosen as a SM-Priority by relatively few patients and rarely emerged as a targeted goal. Finally, none of the 19% of patients who selected taking their medications more regularly as an SM-Priority, a key consideration in chronic disease management, ultimately created an action plan goal in this area. Thus, we note specific SM-Problems were not often being viewed as SM-Priorities, and even among those that did prioritize these areas, rarely were translated into Patient-Healthcare team member goals.

Patient-reported SM-Priorities were in general distributed across the self-management tasks, highlighting the diversity of patient needs. Patients’ selection of SM-Priorities among their SM-Problems might have been influenced by multiple factors related to: (1) individual preferences and/or, (2) perceptions of their healthcare team. Other potential explanations were offered by a recent patient advisory group from five participating primary care clinics. They suggested that patients may be more likely to prioritize a given SM-Problem if they view it as: relatively easy or realistic to tackle, important, particularly pressing, comfortable to discuss, and/or something they have been told or think they “should” work on. Additional contributing patient perceptions of healthcare teams factors may include: patient comfort with the healthcare team member, patient viewing the healthcare team member as competent or potentially positioned to assist given their role or experience, or an area the patient believes is a clinician/healthcare team priority.

Patient centered care and evidence in support of shared decision making emphasizes the importance of patients’ input and voice in care discussions [12, 13]. The lack of concordance between patient SM-Priorities and ultimate action plan goal selection for areas outside of weight loss, diet and exercise may reflect a lack of collaborative decision making driven by healthcare team member lack of knowledge or comfort working with patients around specific areas of self-management. Some healthcare team members may not feel amply trained to work with patients around certain issues, judge that they are best addressed by other members of the healthcare team, or best handled by a referral to a specialist, especially given the individuals selected by practices in the current study (majority in lay healthcare team roles). On the other hand, this lack of concordance could at times reflect healthcare team members’ asserting clinical judgement in concert with patients, in which an action plan veering away from a patient SM-priority could reflect a collaborative process by which the patient and HCP arrive at a new goal together through discussion. Future work that allows for observations of patient-healthcare team member interactions around goal setting in light of SM-Priorities could further contextualize the findings.

The current study has multiple strengths, including a diverse set of health care settings, a diverse group of patients, and a comprehensive yet pragmatic assessment of patient SM-Problems and SM-Priorities. Nonetheless, several limitations are noteworthy. First, patients within clinics were not randomly selected, such that there may have been some bias within clinics regarding patient selection. Second, all variables, including chronic disease status and BMI, were limited to self-report. Confirmation using laboratory and chart data deserve further attention. Third, data presented are limited to only the information entered into the non-EHR integrated CTH program. This did not allow access to clinical referrals or documentation of additional discussions or health plans made with other members of the care team.

## Conclusion

Results of the current study add to our understanding of the multiple and co-occurring SM-Problems and SMS needs that patients along with their healthcare teams s struggle to tackle together. While patients showed great variability in their SM-Priorities regarding self-management tasks, the resulting action plan goals that patients created with their healthcare team member show a striking lack of diversity, with a lopsided focus on weight loss and physical activity at the cost of addressing a diversity of other important patient-reported SM-Problems. There are many reasons why patient SM-Priorities are not ultimately selected as the focus on action plans and further work is needed to understand the likely multifaceted reasons from both patients’ and healthcare team members’ perspectives.

## Data Availability

Data generated and analyzed for the current study will be available from the corresponding author on reasonable request.

## Abbreviations

CTH: Connection to Health
PCMH: Patient-centered medical home
SM-Priorities: Self-management priorities
SM-Problems: Self-management problems
SMS: Self-management support
